# Supplementary material for: Combined NMR and molecular dynamics conformational filter identifies unambiguously dynamic ensembles of Dengue protease NS2B/NS3pro
Source: Commun Biol. 2023 Nov 24;6:1193. doi: 10.1038/s42003-023-05584-6 (PMC10673835; doi:10.1038/s42003-023-05584-6)
Supplement: Supplementary file 2 — Supplementary Information [file 42003_2023_5584_MOESM2_ESM.pdf]

Supplementary Material

Combined NMR and molecular dynamics conformational filter identifies unambiguously dynamic ensembles of Dengue protease NS2B/NS3pro

Tatiana Agback[1#], Dmitry Lesovoy[2,3#], Xiao Han[4], Alexander Lomzov[5], Renhua Sun[4], Tatyana Sandalova[4], Vladislav Yu. Orekhov[3,6], Adnane Achour*[4], Peter Agback*[1]

[1] Department of Molecular Sciences, Swedish University of Agricultural Sciences, PO Box 7015, SE-750 07 Uppsala, Sweden.
[2] Department of Structural Biology, Shemyakin-Ovchinnikov, Institute of Bioorganic Chemistry RAS, 117997, Moscow, Russia
[3] Swedish NMR Centre, University of Gothenburg, Box 465, Gothenburg, 40530 Sweden
[4] Science for Life Laboratory, Department of Medicine, Karolinska Institute, and Division of Infectious Diseases, Karolinska University Hospital, SE-171 76 Stockholm, Sweden.
[5] Laboratory of Structural Biology, Institute of Chemical Biology and Fundamental Medicine SB RAS, 630090, Novosibirsk, Russia.
[6] Department of Chemistry and Molecular Biology, University of Gothenburg, Box 465, Gothenburg, 40530 Sweden.

# These authors contributed equally.
* Corresponding authors: Peter.agback@slu.se, adnane.achour@ki.se

Supplementary Note 1. Four main structural ensembles of NS2B/NS3proS135A were identified based on MD calculations with NMR constraints

MD simulations combined with multiple NMR based constraints are traditionally used to determine the three-dimensional structures of proteins in solution. For small sized folded proteins with rigid architectures, this approach provides most often structural results with very good agreement between X-ray crystallography and NMR spectroscopy approaches. To improve the relaxation properties of the nuclei of larger sized proteins, extensive deuteration of the side chains of amino acid residues is required. This leads often to problems in extracting an adequate number of NMR-based constraints. Moreover, for flexible proteins where the obtained experimental NMR data represent an average of ensembles of conformations, special care should be taken in order to not drive the refined

structure(s) into a one-conformation minima. Keeping this in mind, the aim of the first step of this study was to obtain a limited selection of conformation ensembles that satisfied the limited set of NMR restraints. We therefore chose to use three types of NOE-based restraints including (i) between amide protons (N)H of amino acid residues; (ii) between the amide proton (N)H and side chain methyl protons $(C)H_3$ of Val, Leu and Ile residues, both intra and inter residues, and (iii) between side chain methyl protons $(C)H_3$ of Val, Leu and Ile residues. Altogether, 366 NOE-based restraints were used (**Table S1**). A pool of 334 dihedral angle restraints, predicted by TALOS-N based on the backbone ^{1}HN, ^{15}N, $^{13}C^{\alpha}$, ^{13}CO and sidechain $^{13}C^{\beta}$ chemical shift assignments, previously published by us [1], were used.

Based on these restraints (see the material and methods section for specific descriptions), three main conformational ensembles of NS2B/NS3proS135A were identified with likewise penalties. The domain structures of NS2B/NS3proS135A as a ribbon representation of the final water- and NMR-constrained refined ensembles of 10 NMR structures are presented in **Figure S2**, and annotated as ensemble I* (**Figure S2B**), ensemble II* (**Figure S2C**) and ensemble III* (**Figure S2D**) in which the NS3pro N-terminal his-tag is involved in interactions with the co-factor NS2B. Ensemble I and III have a well-structured β-hairpin formed by residues 76-86 whereas for ensemble II this region is more flexible.

Despite the limited number of restraints for the globular NS3proS135A domain, comprising residues 20–170, our calculations resulted in well-defined structures for each tested initial conformational ensemble. Indeed, root mean square deviation (RMSD) values for the backbone C^{α} atoms of the 10 obtained structures, following cluster analysis

and energy minimization, were 0.97, 1.07 and 1.21 Å for I*, II* and III*, respectively (**Table S2**). Analysis of hydrogen bond interactions formed within NS3pro in the different obtained conformational ensembles for the anti-parallel beta-strands, deduced only from initial calculations from NOEs and dihedral angle restraints, were in agreement with the results from our previous study in which the secondary structure of NS2B/NS3proS135A was predicted using TALOS-N [1]. Each hydrogen bond was within the range of (N)H…O with upper-bound 2.3Å and (H)N…O=C with upper-bound 3.3Å. As expected, the catalytic triad was not formed due to the introduced S135A mutation (**Figure S2**).

Nevertheless, comparison of the structures of the NS3proS135A domain in the different ensembles are varied essentially according to RMSD obtained in the range 1-170 aa on C^{α} measured between the most represented structures of I*, II* and III* ensembles: 1.75, 3.40 and 3.1Å between I*- II*, I*- III* and II*-III*, respectively (**Table S3**). Unsurprisingly, the N- and r C-termini of the NS3proS135A domain are disordered in all three structural ensembles (**Figure S2 B-D**). An unexpected result was found in conformational ensemble III*, in which the N-terminal HIS-tag at one end of the NS3pro domain points towards the C-terminal part of the NS2B co-factor, resulting in specific interactions (**Figure S2D**). The first five N-terminal 43-49 aa residues are disordered (**Figure S2 B-D**) while the following residues 51-57 form a well-defined β-strand. The main differences in the position of the NS2B co-factor in the structural ensembles I*, II* and III* are localized at their C-termini. In the ensemble I*, the secondary structural element formed by residues 75-90 is well defined, consisting of a loop that bridges two β-strands followed by a short α-turn, (**Figure S2B**). In contrast, the C-terminal of NS2B displays in the conformation ensembles II* and III* a significantly larger amount of conformation discrepancies compared to ensemble I*. In conclusion, our analyses

allowed us to identify the unambiguous presence of three conformational ensemble families for the DENV-2 NS2B/NS3proS135A heterodimers that are structurally dispersed, although fulfilling all NMR-based restraints during the performed MD simulations.

Supplementary Note 2. The diffusion tensor of NS2B/NS3proS135A is best fitted with an isotropic tumbling model

The conformational state of NS2B/NS3proS135A was evaluated by experimentally measured ^{15}N-longitudinal relaxation rate (R_1), ^{15}N-transverse relaxation rate (R_2), and heteronuclear Overhauser effects (heteronuclear NOE) (**Figure S7**). A total of 250 resonances were used in the spin relaxation analysis. The residues that were missing or displayed significant broadening were L85, V97, N119, T134, N152, G153, V154, V155, V162, A166 in NS3proS135A, and I76, D81 in NS2B. Analysis performed by Bruker Dynamic centre 2.8 with an anisotropic tumbling model of NS2B/NS3proS135A produced the axially symmetric model with D||/D= 0.907 and the full asymmetry model with Dxx= 7.18e+06(+/- 4.23e+04) s^{-1}, Dyy= 8.37e+06 (+/- 4.43e+04) s^{-1}, Dzz= 7.05e+06 (+/- 3.88e+04) s^{-1}. No significant improvement was observed over the isotropic tumbling model according to an F-test. Isotropic tumbling model analysis yielded an overall correlation time τ_c of 2.08e-08s (with a standard deviation σ = 1.59e-09s) which associates with a monomeric form of NS2B/NS3proS135A in solution. Notably, the aggregation of the protein was negligible in the concentration range stretching from 0.2 to 0.8 mM.

Supplementary Note 3. Molecular dynamic calculations

Molecular models were relaxed in implicit solvent shell using the steepest-descent minimization of 10,000 steps, followed by a conjugate gradient 10,000-step minimization to

remove steric hindrances. Thereafter, the protein molecular models were heated from 0 to 300 K for 100 ps. The Andersen-like temperature coupling scheme was used for temperature regulation[2]. Long-range electrostatics were calculated using the particle mesh Ewald algorithm with a 1Å grid[3]. Hydrogen-containing covalent bonds were constrained using the SHAKE algorithm[4] and time steps of 2fs were used. Thereafter, all molecular models were solvated using a TIP3P water model (cuboid box, distance 12Å)[5] and ionsjc_tip3p parameters for ions[6]. Sodium ions were added into periodic simulation systems to neutralize protein charges. Finally, all molecular models were equilibrated using previously described steps[7].

The models with fixed proteins (constant restraint force of 500 cal/mol/$Å^2$) were minimized using the steepest-descent method with 10,000 steps, followed by the conjugate gradient method comprising 10,000 steps. The system was thereafter heated at a constant volume with fixed proteins for 2.5 ns with time steps of 0.0005ps (constant restraint force of 500 cal/mol/$Å^2$). Next, the system density was equilibrated at a constant pressure of 1 bar and a constant temperature at 300 K (NPT ensemble) for 500ps with fixed proteins (constant restraint force of 500 cal/mol/$Å^2$). Finally, an equilibration step was performed at constant pressure (1 bar) and temperature (300 K) for 5 ns. A Berendsen-type thermostat and barostat were used[8].
Molecular graphics were prepared using UCSF Chimera program v 1.15[9]

Supplementary Note 4. Calculation of theoretical NMR spectral densities from correlation functions

A well-known problem in multi-exponential fitting is the strong sensitivity of the solution to the initial guess of the parameters. For every number of exponents, m, a set of 32+16m fitting runs were used with starting parameter values A_0, A_j, and τ_j chosen according to the assumption that the internal motions take place at significantly different time scales and are uncorrelated [10,11]. While A_0 was calculated as a product of $S^2{}_1 * S^2{}_2 * S^2{}_j * ... * S^2{}_m$ (with $S^2{}_j$ random values from

0 to 1), A_j was calculated as $1-S^2_1$, $S^2_1-S^2_1*S^2_2$ ... $S^2_1*S^2_2*S^2_{j-1}-S^2_1*S^2_2*S^2_j$, whereas τ_j values were randomly chosen from the corresponding intervals ($t_{final}^{j-1/m}$, $t_{final}^{j/m}$). For every m, the solution with the lowest mean square deviation and with $A_0 \geqslant 0$, $A_i \geqslant 0$, $\tau_i \geqslant 0$ was chosen. A total of six solutions were obtained (m=2…7), and the final solution was chosen according to the criteria in which the mean square deviation was significantly reduced with increased number of exponents, *i.e.* along the line of the Bayesian information criterion (BIC). The starting parameters for cross-correlation function approximation were similar, although with S^2_j random values from -1 to 1 with $A_0=S^2_1*S^2_2*S^2_j*…*S^2_m$, and the starting A_j values were calculated as $P_2(\cos\theta_{HXH'})-S^2_1$, $S^2_1-S^2_1*S^2_2$ ... $S^2_1*S^2_2*S^2_{j-1}-S^2_1*S^2_2*S^2_j$, where the results were selected by criteria $\tau_j \geqslant 0$.

Supplementary Figures

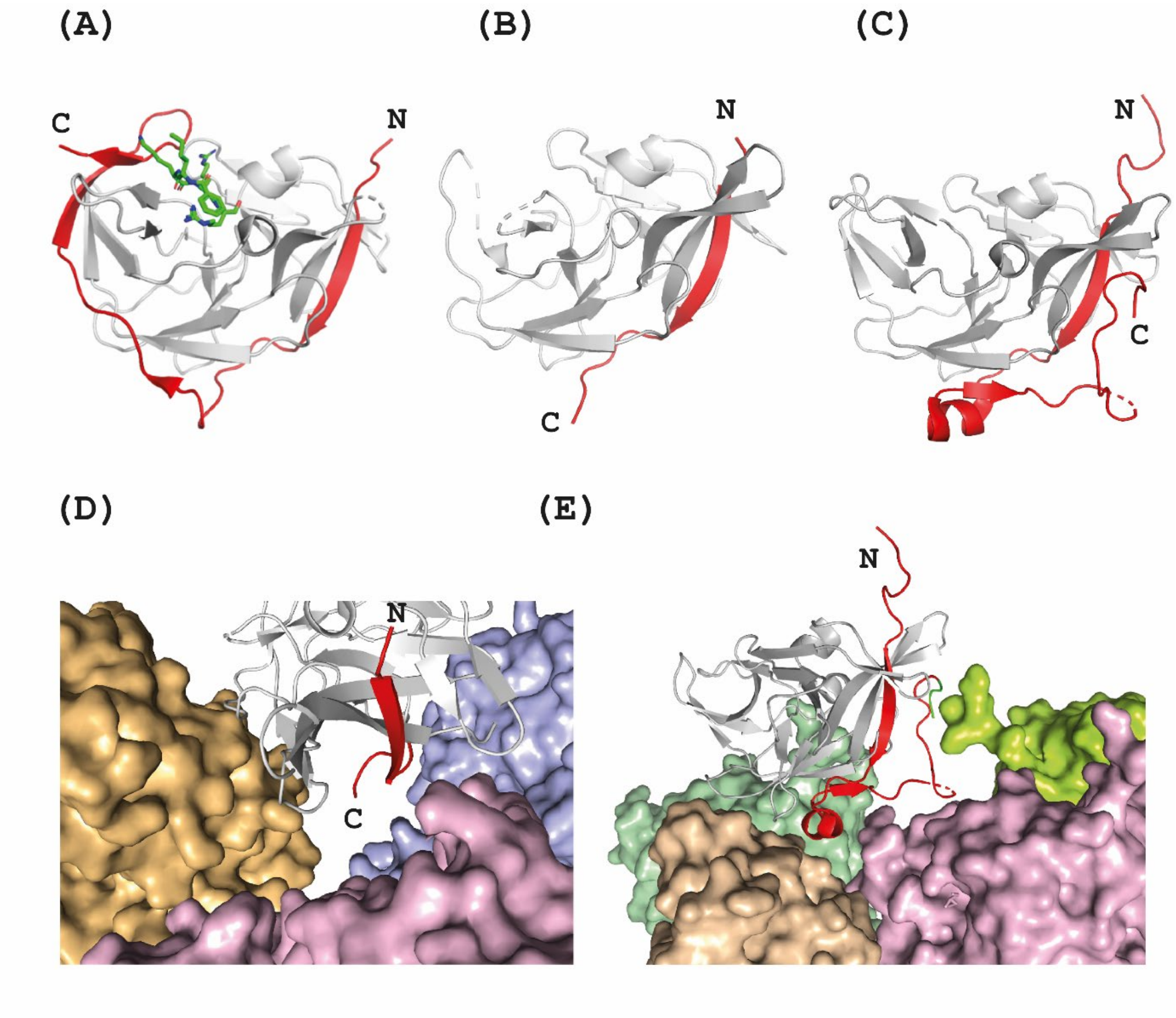

Figure S1. Different conformations of NS2B (in red) when bound to NS3pro (in grey)
(A) ‘Closed’ conformation taken by NS2B in the DENV-2 NS2B/NS3pro complex. An inhibitor bound in the active site of protease is coloured in green (PDB code 2FP7). **(B)** Disordered ‘open’ conformation of NS2B in the ligand-free form of the DENV-4 NS2B/NS3pro complex (PDB code 5YVJ). The NS2B residues 62-96 are not visible in the electron density. **(C)** NS2B takes an alternative ‘open’ conformation in the ligand-free DENV-2 NS2B/NS3pro protease complex (PDB code 2FOM). **(D)** The NS2B stretch of residues 62-96, which is not visible in the crystal structure of DENV-4 NS2B/NS3pro, are localized in a cavity formed between symmetry-related molecules, each shown as a coloured surface (PDB code 5YVJ). **(E)** The C-terminus of NS2B in the DENV-2 NS2B/NS3pro complex forms close contacts with symmetry-related molecules, which are shown as surfaces coloured in different colours. These close contact interactions may induce an alternative ”open” conformation (PDB code 2FOM).

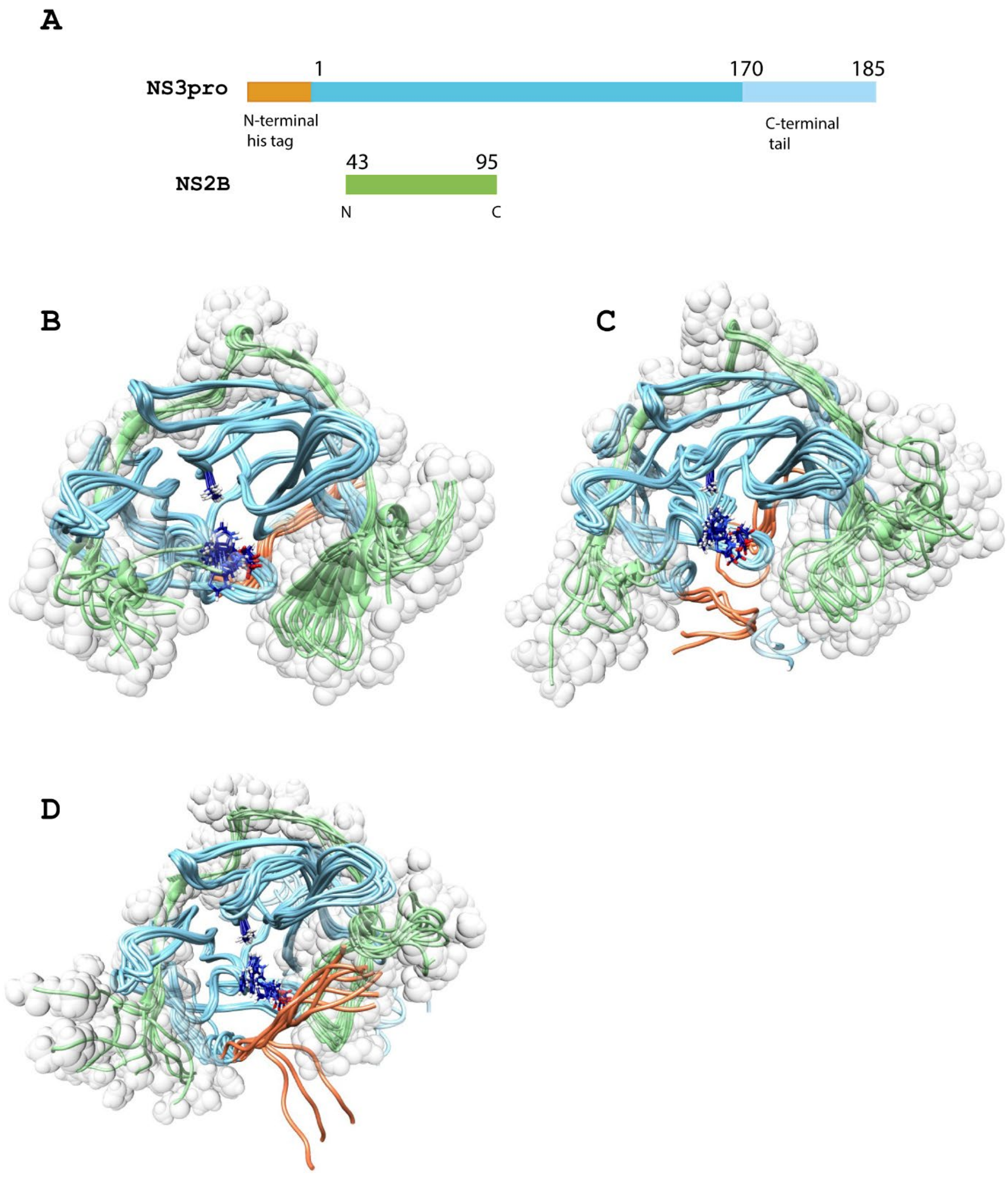

Figure S2. Ensembles of solution NMR structures of the DENV-2 NS2B/NS3proS135A heterodimer obtained through long 1μs MD simulations. (**A**) The schematic colours sequences of the NS3proS135A and NS2B domains are displayed with corresponding numbering and used in the structural models presented in (**B**)-(**D**). Domain structure and ribbon representation of the final water- and NMR-constrained refined ensembles of 10 NMR structures of the DENV-2-associated NS2B/NS3proS135A mutated variant are presented, including (**B**), (**C**) and (**D**) I*, II* and III*. All three ensembles were thereafter minimized using the steepest-descent minimization approach based 100000-steps, followed by a conjugate gradient of 100000-step up to the reaching minimum. The NMR restraints (Table S1) were applied at minimization stage.

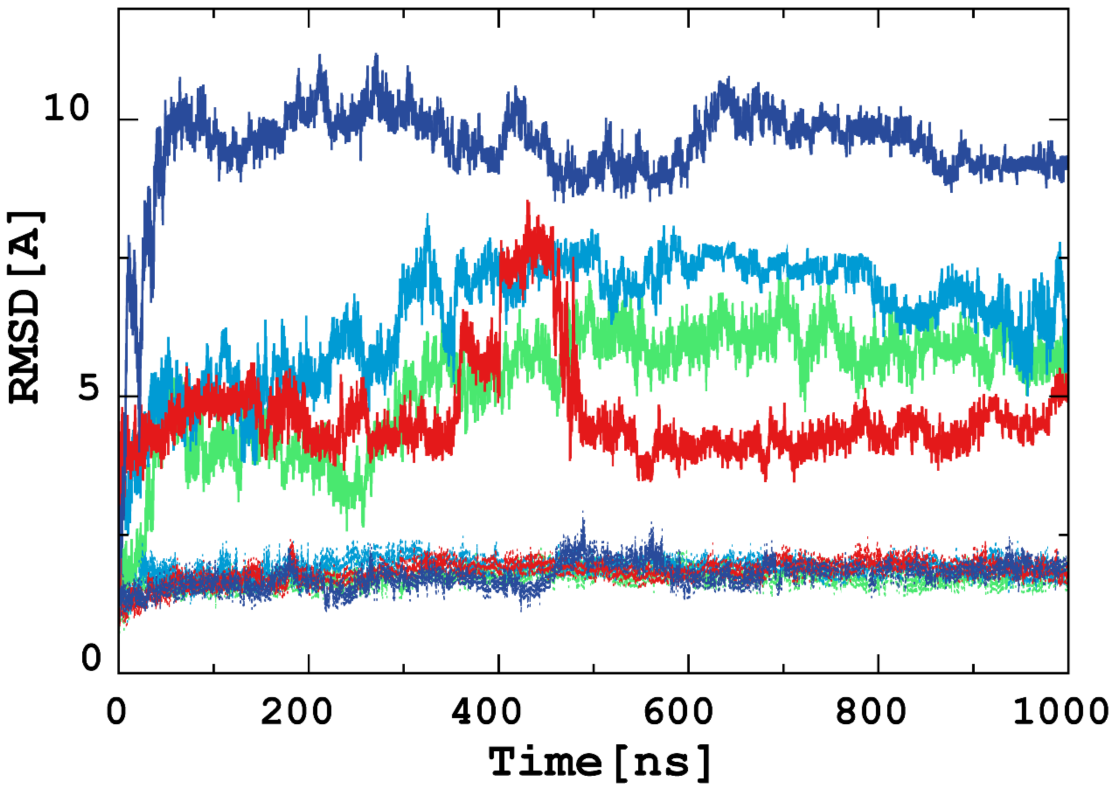

Figure S3 The RMSD of the heavy backbone atoms calculated for 1 μs free restraints MD simulations for DENV-2 NS2B/NS3proS135A. Values for ensembles I, II, III and IV shown in green, light blue, red and dark blue, respectively. Thick lines corresponded to the all residues of the complex, thin dash lines - for dynamically stable residues 20-168 of NS3proS135A and 51-72 NS2B.

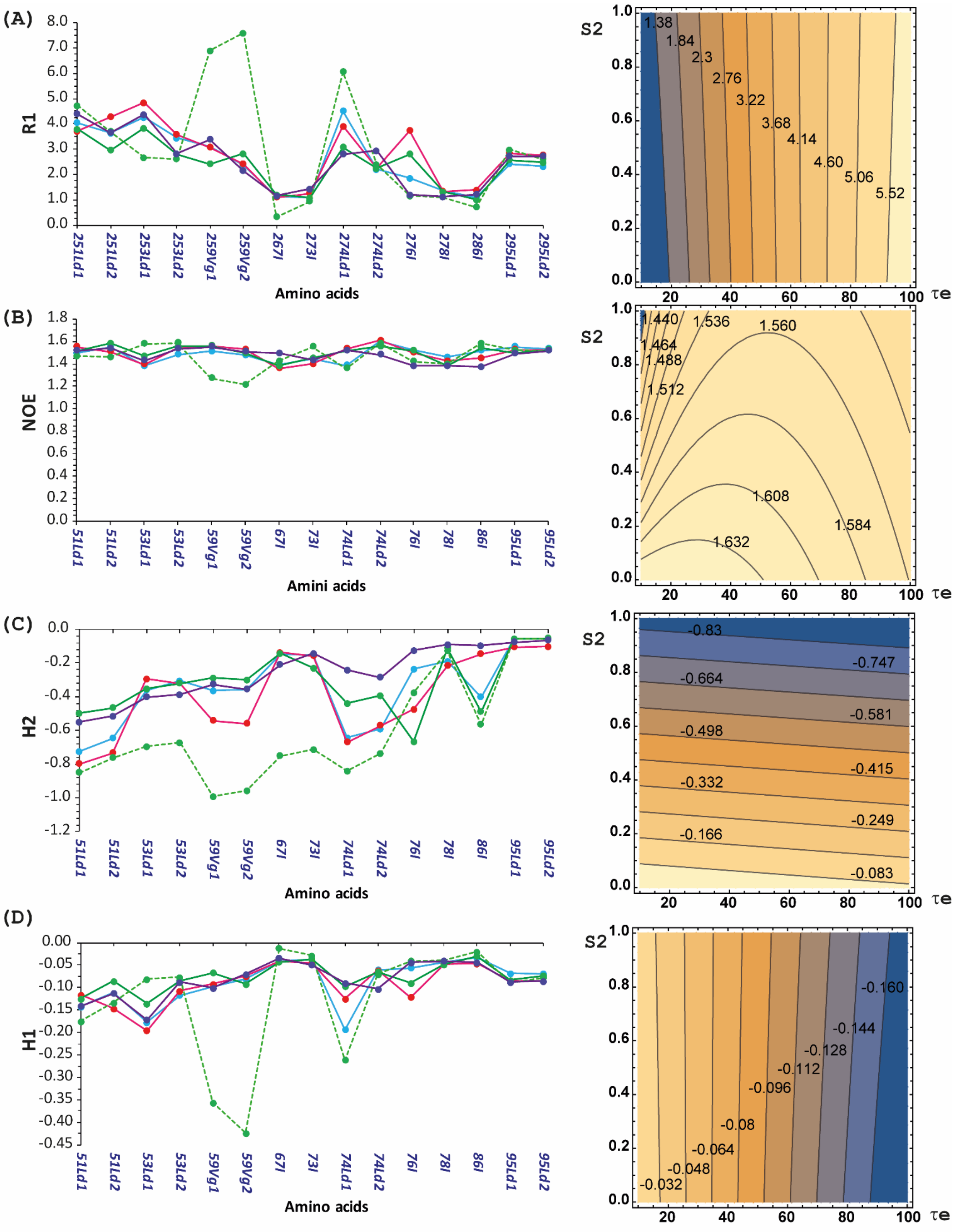
(A)
R1
8.0
7.0
6.0
5.0
4.0
3.0
2.0
1.0
0.0
251Ld1
251Ld2
253Ld1
253Ld2
259Vg1
259Vg2
267I
273I
274Ld1
274Ld2
276I
278I
286I
295Ld1
295Ld2
Amino acids
S2
1.38
1.84
2.3
2.76
3.22
3.68
4.14
4.60
5.06
5.52
τe
(B)
NOE
1.8
1.6
1.4
1.2
1.0
0.8
0.6
0.4
0.2
0.0
51Ld1
51Ld2
53Ld1
53Ld2
59Vg1
59Vg2
67I
73I
74Ld1
74Ld2
76I
78I
86I
95Ld1
95Ld2
Amini acids
1.440
1.536
1.560
1.464
1.488
1.512
1.608
1.584
1.632
(C)
H2
0.0
-0.2
-0.4
-0.6
-0.8
-1.0
-1.2
Amino acids
-0.83
-0.747
-0.664
-0.581
-0.498
-0.415
-0.332
-0.249
-0.166
-0.083
(D)
H1
0.00
-0.05
-0.10
-0.15
-0.20
-0.25
-0.30
-0.35
-0.40
-0.45
Amino acids
-0.160
-0.144
-0.128
-0.112
-0.096
-0.08
-0.064
-0.048
-0.032
20
40
60
80
100

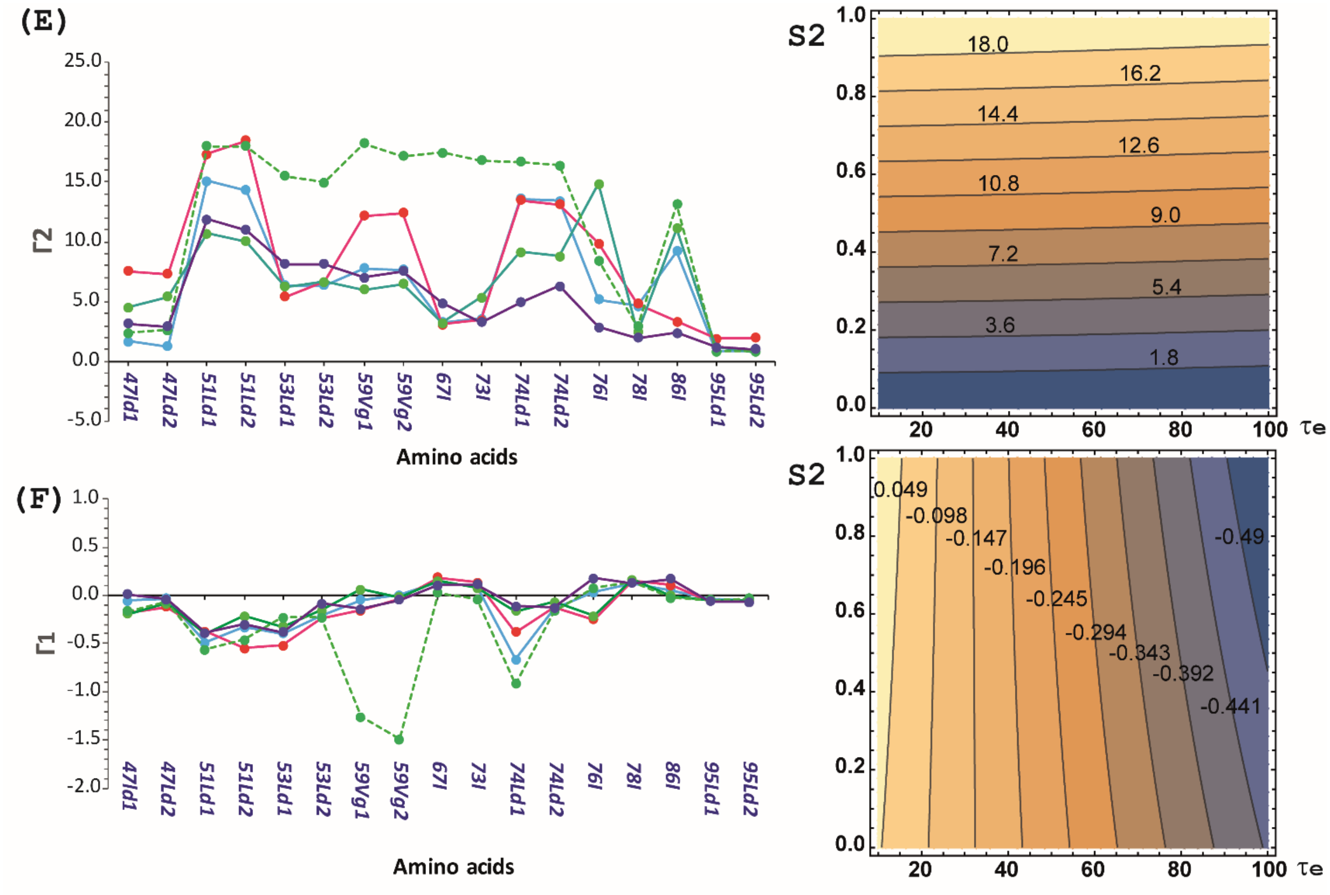

Figure S4. Simulated relaxation parameters for methyl groups in the different conformation ensembles of the NS2B co-factor obtained from free restraints MD simulations

Relaxation rates simulated from different trajectories (**left panels**) and their theoretical profiles (**right panels**) as a function of internal motion τ_e and amplitude S^2 are presented in (**A**) longitudinal relaxation rate R_1, (**B**) heteronuclear ^1H-^{13}C NOE, (**C**) H1 and (**D**) H2 which are CSA/dipolar cross-correlation contribution to longitudinal R_1 and transverse R_2 relaxation rates, respectively. Finally, the CH-CH dipolar cross-correlations to transverse Γ2 and longitudinal Γ1 relaxation rates are presented in panels **(E)** and **(F)**, respectively. The theoretically predicted dynamic parameters R_1, R_2 and NOE, obtained through measurements of five trajectories are shown by solid lines for each of the different initial ensembles of structural conformations, I (green), II (light blue), III (red), IV (dark blue) and V (green dashed).The S^2 and τ_e parameters are annotated as described in **Figure S6**. Relaxation rate values were calculated assuming isotropic tumbling of NS2B/NS3proS135A with an overall correlation time of 20.8ns and a proton Larmor frequency of 800MHz.

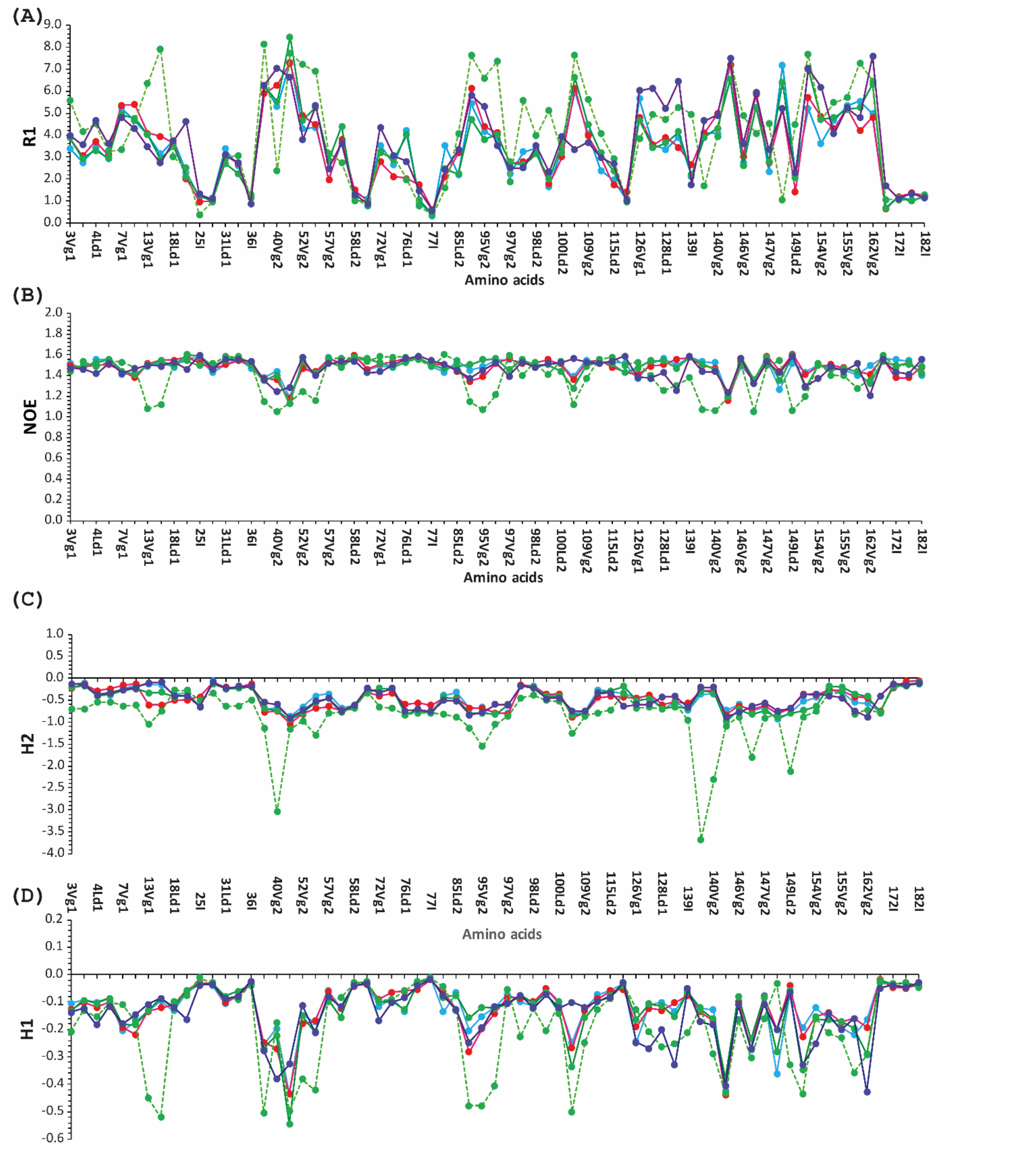
(A)
R1
Amino acids
(B)
NOE
Amino acids
(C)
H2
(D)
Amino acids
H1
3Vg1
4Ld1
7Vg1
13Vg1
18Ld1
25I
31Ld1
36I
40Vg2
52Vg2
57Vg2
58Ld2
72Vg1
76Ld1
77I
85Ld2
95Vg2
97Vg2
98Ld2
100Ld2
109Vg2
115Ld2
126Vg1
128Ld1
139I
140Vg2
146Vg2
147Vg2
149Ld2
154Vg2
155Vg2
162Vg2
172I
182I

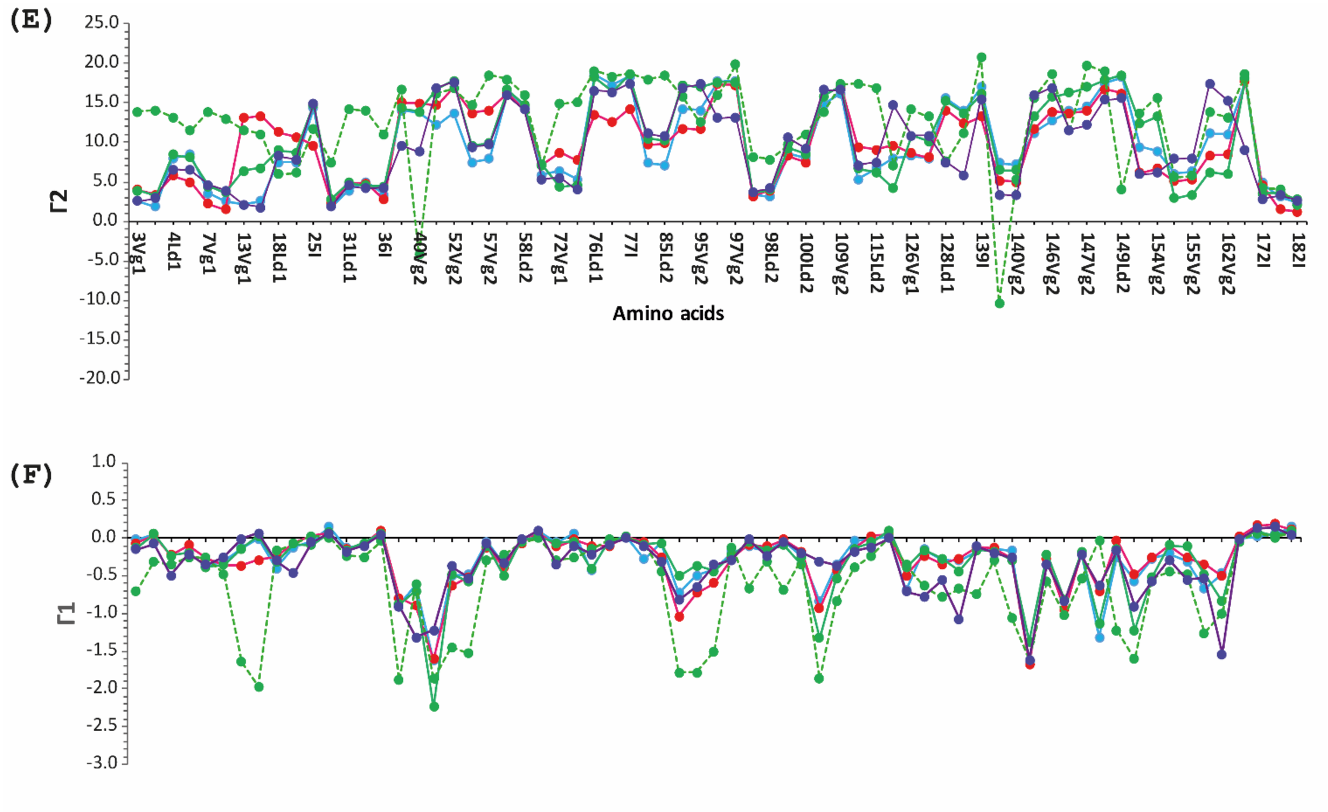

Figure S5. Simulated relaxation parameters of methyl groups for NS3proS135A among the different identified the conformational ensembles obtained from free restraints MD simulations

Relaxation rates simulated from different trajectories are presented, including (**A**) longitudinal relaxation rate R_1, (**B**) heteronuclear ^{1}H-^{13}C NOE, (**C**) H1and (**D**) H2 which are the CSA/dipolar cross-correlation contributions to longitudinal R_1 and transverse R_2 relaxation rates, respectively. The CH-CH dipolar cross-correlations to transverse Γ2 and longitudinal Γ1 relaxation rates are presented in (**E**) and (**F**), respectively. Conformation ensembles I, II, III, IV and V were green, blue, red, dark blue and dashed green, respectively. Relaxation rate values were calculated assuming an isotropic tumbling of NS2B/NS3proS135A with an overall correlation time τ_c of $2.08e^{-08}$ s and a proton Larmor frequency of 800MHz. S^2 and τ_e parameters are annotated as described in **Figure S6**.

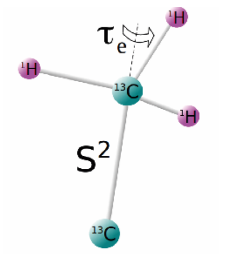

Figure S6. Simple motional model for a methyl group

A molecular model of a methyl group includes simultaneous fast free rotation about the C-C' axis with a correlation time τ_e, and relatively slow motion of the C-C' axis itself. The corresponding auto correlation order parameter of the C-C' axis is S^2 [12].

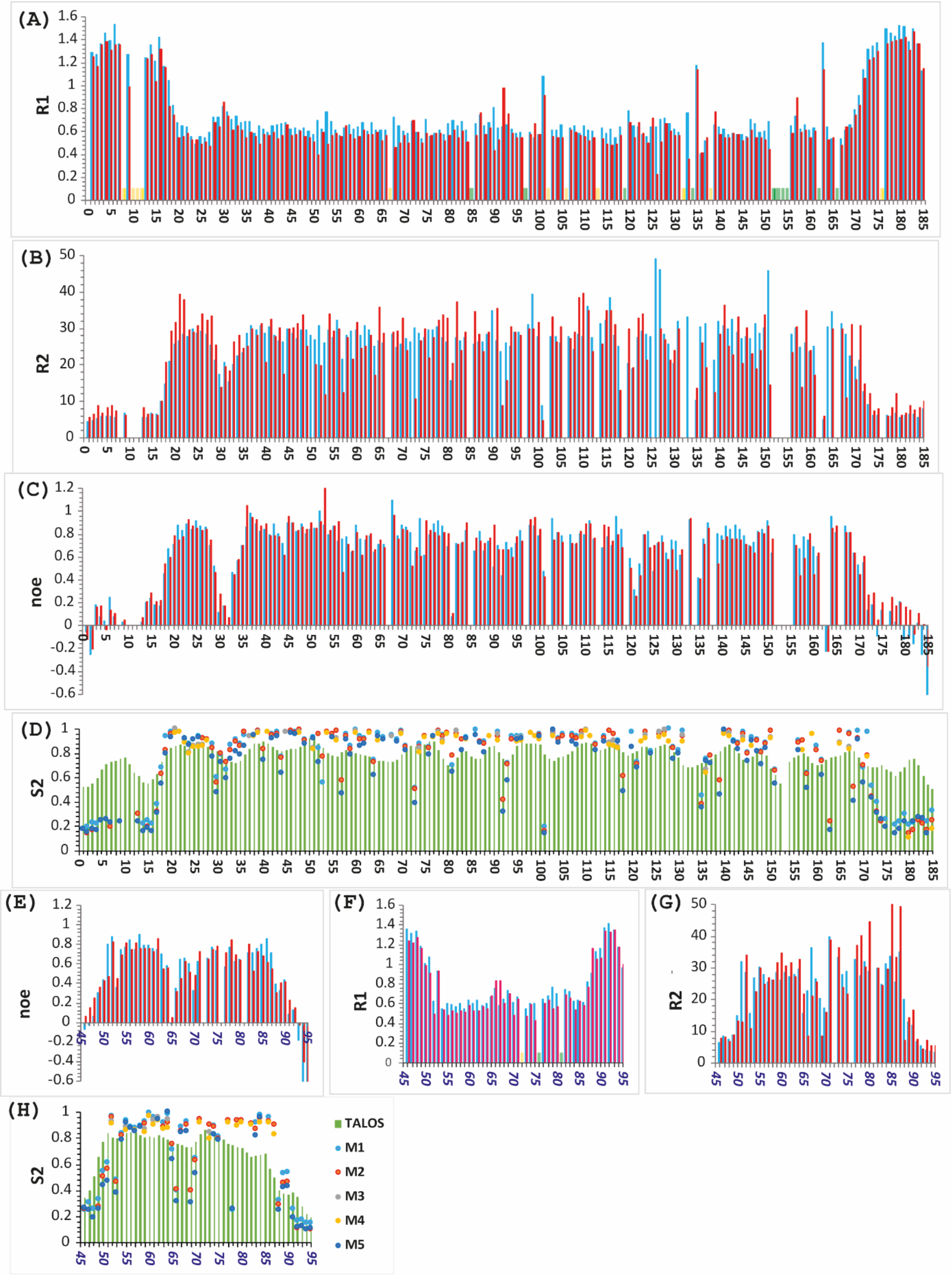

Figure S7. Dynamic parameters for NS3proS135A (A-D) and NS2B (E-L) obtained at 600 and 700MHz frequencies. The longitude relaxation rate $R_1(s^{-1})$ is presented for each residue of NS3pro (**A**) and NS2B (**F**) domains. The transverse relaxation time $R_2(s^{-1})$ is also presented for NS3proS135A (**B**) and NS2B (**G**). The NOE values for NS3proS135A and NS2B are presented in (**C**) and (**E**), respectively. The experimentally obtained $R_1(s^{-1})$, $R_2(s^{-1})$ and NOE are represented

by blue and red solid brackets for values obtained at 600 and 700MHz frequencies, respectively. The S2 order parameters, extracted from our experimental data, R_1, R_2 and NOE, are presented for NS3proS135A and NS2B in (**D**) and (**H**), respectively. Five molecular models free analyse[13,14] M1-M5, were used and the results are presented by circles with different colour, allowing for comparison with results from TALOS predictions. The identity of the different colours is described on the right side of Panel (**L**). The TALOS-N-predicted S2 order parameters, based on chemical shifts of backbone nuclei, are presented by green solid brackets (**D**) and (**H**). Proline and unassigned amino acid NH resonances are shown by short yellow and green boxes, respectively, in panels (**A**) and (**F**).

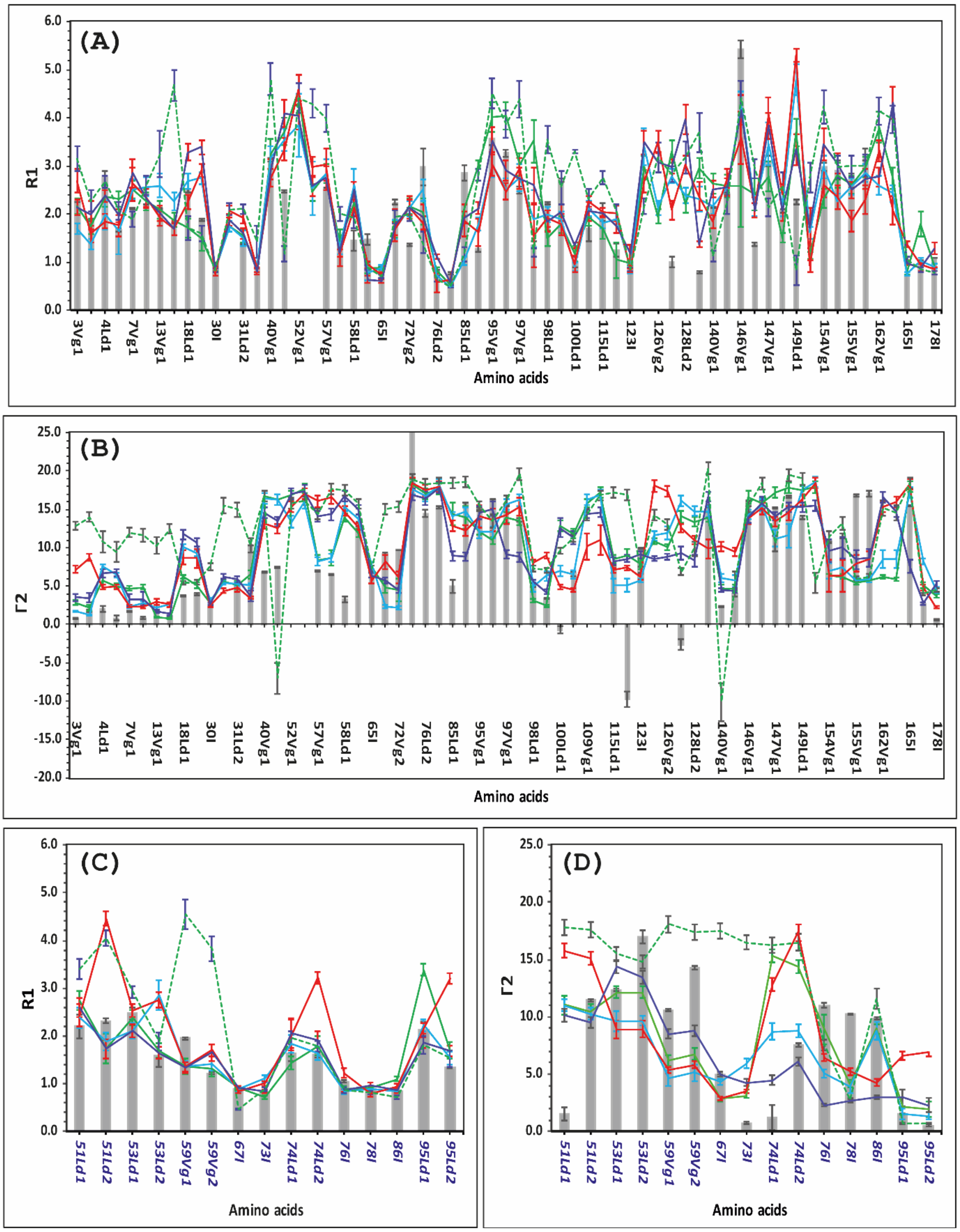

Figure S8. DENV-2 S135A Methyl dynamical parameters of the NS3proS135A (A, B) and co factor NS2B (C, D) obtained on 800MHz spectrometers

For NS3proS135A and NS2B the relaxation rate values $R_1(s^{-1})$ and $\Gamma 2(s^{-1})$ are presented in panels (**A**), (**C**) and (**B**), (**D**), respectively. The experimentally obtained relaxation time $R_1(s^{-1})$ and $\Gamma 2(s^{-1})$ for NS3proS135A and NS2B are presented by grey solid brackets. The theoretically predicted dynamical parameters $R_1(s^{-1})$ and $\Gamma 2(s^{-1})$, derived from five trajectories, are shown by solid lines, coloured in green, light blue, red, dark blue and dashed green line for the conformational ensembles I, II, III; IV and V, respectively.

Table S1. NMR and Refinement Statistics for the NS2B/NS3proS135A heterodimer

	NS2B/NS3proS135A
**Distance restraints**:	
total NOE	366
Intra-residue	59/14
inter-residue<br>sequential ($\|i - j\| = 1$)	141/31
medium-range ($\|i - j\| \leq 4$)	38/1
long-range ($\|i - j\| \geq 5$)	62/1
intermolecular restraints	19
sequential NH-NH	99/24
Inter strand NH-NH	9
**Dihedral restraints**	
total dihedral restraints	334
ϕ	140/27
ψ	140/27

Table S2. RMSD values in ensembles of 10 structures obtained by cluster analysis of 1 μs NMR-restrained trajectory and minimization

Description	Ensemble	RMSD values for NS3proS135A, residues 1–170 for C_α atoms, Å	RMSD values for NS3proS135A, residues 20–170 for C_α atoms, Å
closed conformation	I*	1.02	0.97
partly opened	II*	2.14	1.07
partly opened- tag	III*	1.39	1.21
**Average**		1.52	1.08

Table S3. RMSD values between the most representative structures obtained by cluster analysis from a 1 μs NMR-restrained trajectory and minimization.

Description	Used annotation	RMSD values for NS3proS135A residues 1–170 for C-alpha atoms, Å	RMSD values for NS3proS135A residues 20–170 for C-alpha atoms, Å
closed conformation vs partly opened	I* vs II*	1.75	1.03
closed conformation vs partly opened-tag	I* vs III*	3.40	1.38
partly opened vs partly opened-tag	II* vs III*	3.10	0.84
**Average**		**2.75**	**1.08**

Supplementary References

1 Agback, P. *et al.* H-1, C-13 and N-15 resonance assignment of backbone and IVL-methyl side chain of the S135A mutant NS3pro/NS2B protein of Dengue II virus reveals unique secondary structure features in solution. *Biomolecular Nmr Assignments* **16**, 135-145 (2022). https://doi.org:10.1007/s12104-022-10071-w

2 Andersen, H. C. Molecular-Dynamics Simulations at Constant Pressure and-or Temperature. *Journal of Chemical Physics* **72**, 2384-2393 (1980). https://doi.org:Doi 10.1063/1.439486

3 Darden, T., York, D. & Pedersen, L. Particle Mesh Ewald - an N.Log(N) Method for Ewald Sums in Large Systems. *Journal of Chemical Physics* **98**, 10089-10092 (1993). https://doi.org:Doi 10.1063/1.464397

4 Miyamoto, S. & Kollman, P. A. Settle - an Analytical Version of the Shake and Rattle Algorithm for Rigid Water Models. *Journal of Computational Chemistry* **13**, 952-962 (1992). https://doi.org:DOI 10.1002/jcc.540130805

5 Jorgensen, W. L., Chandrasekhar, J., Madura, J. D., Impey, R. W. & Klein, M. L. Comparison of Simple Potential Functions for Simulating Liquid Water. *Journal of Chemical Physics* **79**, 926-935 (1983). https://doi.org:Doi 10.1063/1.445869

6 Joung, I. S. & Cheatham, T. E. Determination of alkali and halide monovalent ion parameters for use in explicitly solvated biomolecular simulations. *J Phys Chem B* **112**, 9020-9041 (2008). https://doi.org:10.1021/jp8001614

7 Lomzov, A. A., Vorobjev, Y. N. & Pyshnyi, D. V. Evaluation of the Gibbs Free Energy Changes and Melting Temperatures of DNA/DNA Duplexes Using Hybridization Enthalpy Calculated by Molecular Dynamics Simulation. *J. Phys. Chem. B* **119**, 15221–15234 (2015). https://doi.org:doi.org/10.1021/acs.jpcb.5b09645

8 Berendsen, H. J. C., Postma, J. P. M., Vangunsteren, W. F., Dinola, A. & Haak, J. R. Molecular-Dynamics with Coupling to an External Bath. *Journal of Chemical Physics* **81**, 3684-3690 (1984). https://doi.org:Doi 10.1063/1.448118

9 Pettersen, E. F. *et al.* UCSF chimera - A visualization system for exploratory research and analysis. *Journal of Computational Chemistry* **25**, 1605-1612 (2004). https://doi.org:10.1002/jcc.20084

10 Ferrage, F. & Dorai, K. in *Cross-relaxation and Cross-correlation Parameters in NMR: Molecular Approaches* (ed D. Canet) Ch. 4, 239-315 (Royal Society of Chemistry, 2018).

11 Yang, D. W. Probing Protein Side Chain Dynamics Via C-13 NMR Relaxation. *Protein Peptide Lett* **18**, 380-395 (2011). https://doi.org:Doi 10.2174/092986611794653932

12 Zhang, X., Sui, X. G. & Yang, D. W. Probing methyl dynamics from C-13 autocorrelated and cross-correlated relaxation. *J Am Chem Soc* **128**, 5073-5081 (2006). https://doi.org:10.1021/ja057579r

13 Lipari, G. & Szabo, A. Model-Free Approach to the Interpretation of Nuclear Magnetic-Resonance Relaxation in Macromolecules .2. Analysis of Experimental Results. *J Am Chem Soc* **104**, 4559-4570 (1982). https://doi.org:DOI 10.1021/ja00381a010

14 Lipari, G. & Szabo, A. Model-Free Approach to the Interpretation of Nuclear Magnetic-Resonance Relaxation in Macromolecules .1. Theory and Range of Validity. *J Am Chem Soc* **104**, 4546-4559 (1982). https://doi.org:DOI 10.1021/ja00381a009
